# Polymorphic variants of *IGF2BP3* and *SENCR* have an impact on predisposition and/or progression of Ewing sarcoma

**DOI:** 10.3389/fonc.2022.968884

**Published:** 2022-10-21

**Authors:** Marcella Martinelli, Caterina Mancarella, Luca Scapoli, Annalisa Palmieri, Paola De Sanctis, Cristina Ferrari, Michela Pasello, Cinzia Zucchini, Katia Scotlandi

**Affiliations:** ^1^ Department of Experimental, Diagnostic and Specialty Medicine, University of Bologna, Bologna, Italy; ^2^ Laboratory of Experimental Oncology, IRCCS Istituto Ortopedico Rizzoli, Bologna, Italy

**Keywords:** Ewing sarcoma, polymorphisms, *IGF2BP3*, *SENCR*, cancer predisposition

## Abstract

Ewing sarcoma (EWS), the second most common malignant bone tumor in children and adolescents, occurs abruptly without clear evidence of tumor history or progression. Previous association studies have identified some inherited variants associated with the risk of developing EWS but a common picture of the germline susceptibility to this tumor remains largely unclear. Here, we examine the association between thirty single nucleotide polymorphisms (SNPs) of the *IGF2BP3*, a gene that codes for an oncofetal RNA-binding protein demonstrated to be important for EWS patient’s risk stratification, and five SNPs of *SENCR*, a long non-coding RNA shown to regulate *IGF2BP3*. An association between polymorphisms and EWS susceptibility was observed for three *IGF2BP3* SNPs - rs112316332, rs13242065, rs12700421 - and for four *SENCR* SNPs - rs10893909, rs11221437, rs12420823, rs4526784 -. In addition, *IGF2BP3* rs34033684 and *SENCR* rs10893909 variants increased the risk for female respect to male subgroup when carried together, while *IGF2BP3* rs13242065 or rs76983703 variants reduced the probability of a disease later onset (> 14 years). Moreover, the absence of *IGF2BP3* rs10488282 variant and the presence of rs199653 or rs35875486 variant were significantly associated with a worse survival in EWS patients with localized disease at diagnosis. Overall, our data provide the first evidence linking genetic variants of *IGF2BP3* and its modulator *SENCR* to the risk of EWS development and to disease progression, thus supporting the concept that heritable factors can influence susceptibility to EWS and may help to predict patient prognosis.

## Graphical Abstract

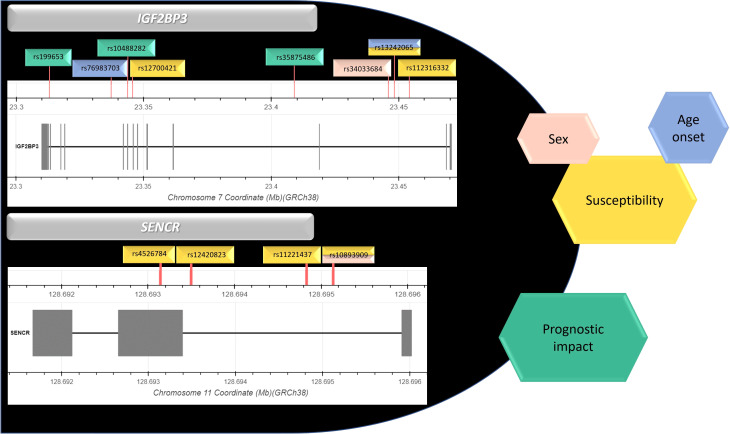

## Introduction

Ewing sarcoma (EWS), the second most common primary tumor of bone in the pediatric population, is a mesenchymal very aggressive cancer with high tendency to form distal metastasis and still unmet clinical needs (1). From a genetic point of view, EWS is characterized by a very low mutational burden (2–4) while its genetic landscape is thought to be driven by the aberrant transcript that derives from the fusion of *EWSR1* gene with a member of the *ETS* family genes, in most of the cases represented by *EWSR1-FLI* chimera (5). EWS is not considered a heritable cancer but disparity in EWS epidemiological distribution, with higher incidence in European than in Asian and African population (6) together with some reports indicating EWS in siblings or cousins (7, 8) and reports of family aggregation of different malignant tumors between EWS patients and their relatives (9, 10) suggest that genetic susceptibility factors may exist for this tumor. Indeed, in the last decade several evidence of correlation between polymorphic variants and EWS risk has been reported (11–18). Through genome-wide association studies (GWAS), multiple genetic susceptibility loci (1p36.22, 6p25.1, 10q21, 15q15, 20p11.22 and 20p11.23) associated with EWS risk have been identified (16, 17). Most of these loci reside near GGAA repeat sequences and may condition the binding of the aberrant transcriptional factor EWS-FLI1. A noteworthy example is the locus 10q21, in which rs79965208 variant increases the number of consecutive GGAA motifs and the consequent EWS-FLI1-dependent enhancer activity, leading to *EGR2* overexpression and favoring EWS susceptibility and aggressiveness (18). Deeply investigation of genes already known to be involved in the pathogenesis and progression of EWS has been performed by several groups as an alternative option to identify predisposing factors for this disease. In particular, analysis of single nucleotide polymorphisms (SNPs) in the *EWSR1* gene revealed that the rs4820804 variant in homozygosis may increase the chance of chromosome breakage and occurrence of chromosomal translocation (11). The relationship between polymorphic variants in genes implicated in EWS pathogenesis and progression and their role in EWS susceptibility has been also studied for *NROB1* and *CAV1*, two EWS-FLI1 target genes (19), and for *CD99*, another hallmark of EWS critically associated with EWS cell differentiation, migration and metastasis (20). Specifically, *CD99* rs311059-T allele was found to be associated with early EWS onset, while rs312257-T variant was related with a reduced risk of relapse (12). In this study, we focused on the analysis of genetic polymorphisms of the Insulin-like growth factor 2 mRNA-binding protein 3 (*IGF2BP3*), a gene that encodes for an oncofetal RNA-binding protein that was found to be undetectable in most adult tissues but strongly expressed in embryos and in diverse tumor types (21). In EWS, high levels of IGF2BP3 were found to support cell migration and metastasis formation besides correlating with disease progression and poor patient’s prognosis (22–24). Thirty SNPs mapping on *IGF2BP3* were genotyped in a cohort of 73 EWS Italian patients to evaluate the genetic influence of *IGF2BP3* polymorphisms on EWS susceptibility and to establish whether a potential link between *IGF2BP3* somatic variants and EWS progression exists. In addition, we analyzed five genetic polymorphisms of *SENCR*, a long non-coding RNA transcribed antisense from the 5’ end of the *FLI1* gene, which was shown to regulate IGF2BP3 (25).

## Materials and methods

### Patients and control group

A cohort of 73 unrelated Italian patients with localized (58 cases) or disseminated (15 cases) EWS treated at the IRCCS Istituto Ortopedico Rizzoli (Bologna, Italy) was considered. Patients underwent local treatments (surgery; surgery plus radiotherapy; radiotherapy only, when the surgeon considered the lesion inoperable or due to patient refusal) and neo-adjuvant chemotherapy according to protocols that were previously reported in detail (26, 27). For radiotherapy administered in combination with surgery, the doses ranged between 45 Gy and 54 Gy, depending on the individual factors (age, site, size, surgical margins, response to chemotherapy); for radiotherapy administered alone, the doses ranged between 54-60 Gy. The timing of radiation therapy ranged between 4 to 6 weeks (26, 27). Clinical-pathological data are shown in **Table 1**. Patients with localized EWS were followed-up for 120 months and clinical information updated. Ethical committee approval was obtained from the Comitato Etico di Area Vasta Emilia Centro (Codice CE AVEC 505/2019/Sper/IOR) and written informed consent was obtained. The study was conducted in accordance with the Declaration of Helsinki ethical guidelines. Control sample consisted of three populations among the 1000 Genome Project, i.e Toscani in Italia (TSI), Utah residents with Northern and Western European ancestry (CEU), and Iberian population in Spain (IBS). Genotypes for each polymorphism were obtained from the Ensembl.org genome browser (GRCh37/hg19).

**Table 1:** Clinical-pathological features of EWS patients.

	Dataset I (N = 73)		Dataset II (N = 58)	
Characteristics	N	%	N	%
**Gender**				
Female	23	31.5	20	34.5
Male	50	68.5	38	65.5
**Age**				
≤ 14 years	11	15.1	10	17.2
> 14 years	62	84.9	48	82.8
**Location**				
Extremity	47	64.4	40	69
Central	12	16.4	7	12
Pelvis	14	19.2	11	19
**Metastasis at diagnosis**				
Yes	15	20.5	0	0
No	58	79.5	58	100
**Local Treatment**				
RxT	10	13.7	7	12.1
RxT+Surgery	24	32.9	18	31
Surgery	39	53.4	33	56.9
**Surgical Treatment**				
Resection	58	92	46	90.2
Amputation	5	8	5	9.8
**Surgical Margins**				
Radical	1	1.6	1	2
Wide	52	82.5	43	84.3
Marginal	7	11.1	6	11.7
Intralesional	3	4.8	1	2
**OVS (Status)**				
Alive	36	49.3	35	60.3
Dead	37	50.7	23	39.7

Dataset I includes primary localized and metastatic tumors at diagnosis; Dataset II includes only primary localized lesions.

OVS, overall survival.

### Single nucleotide polymorphism genotyping

Quality and concentration of DNA obtained from peripheral blood leukocytes or from muscle tissue using standard DNAzol procedure (Thermo Fisher Scientific, Foster City, CA, USA) were evaluated by Nanodrop (Thermo Fisher Scientific). Aliquots of 12 ng/μl DNA from each patient were plated for being processed by the Sequenom MALDI-TOF mass spectrometer MassArray system (as a service at Applied Biomedical Research Center, S. Orsola-Malpighi Polyclinic, Bologna, Italy). The SNPs were selected using the SNPclip tool [https://ldlink.nci.nih.gov/] among the Genome1000 Phase1 Vars (GRCh37/hg19). Caucasian, Italian and Iberian populations (CEU+TSI+IBS dataset) were used to explore the haplotype complexity of each locus considered, applying the thresholds R^2^ 0.8 and MAF 0.07. To a selection of 30 SNPs distributed along the entire *IGF2BP3* sequence with the minimal redundancy level (28) were added 5 SNPs of the *SENCR* gene. Assay design was performed using specific Sequenom software package (Sequenom, San Diego, California, USA). Primers were synthesized at Metabion (Martinsried, Germany) (sequences available upon request). Allele peaks were analyzed with the Sequenom Typer Analysis software.

### Statistical analysis

The distribution of genotypes in patient and control groups was tested for deviations from the Hardy–Weinberg equilibrium using Pearson’s χ^2^ test. The PLINK software was used to test for allelic association within different sample subsets, defined by patient sex or age at disease onset within an alternate phenotype file (29). Odds ratios were calculated to estimate the level of association of the rare allele carriers, i.e heterozygotes versus non-carriers, as well as homozygotes versus non-carriers. A permutation procedure was used to generate empirical significance levels. Such procedure relaxed assumptions about normality of continuous phenotypes and Hardy-Weinberg equilibrium and faced with rare alleles and small sample sizes. Haplotype association analysis using a likelihood ratio approach was performed with the aid of UNPHASED software v3.1.7 for loci showing nominal evidence of association in allelic association analysis. The full model test was performed to obtain a global *P* for association. In addition, for the combinations of SNPs that showed a global *P* < 0.05 in the overall association test, a specific analysis was carried out to evaluate a difference in risk between one haplotype versus all the others pooled together.

Association between *IGF2BP3* or *SENCR* variants and overall survival (OVS) was also estimated. Survival curves for OVS were obtained using the Kaplan–Meier method, while the log-rank test was used to calculate univariate statistical significance. OVS was defined as the time from diagnosis to cancer-related death. Patients who were lost to follow-up were censored at the last contact date. The genetic variants significantly associated with OVS in univariate analysis were entered into a Cox proportional hazards model. Values of 95% confidence intervals (CIs) of hazard ratios (HRs) were provided (30). Analyses were performed with SPSS software, version 22.0. *P* value ≤ 0.05 were considered significant.

## Results

### Impact of the *IGF2BP3* and *SENCR* SNPs on the risk to develop EWS

Genotyping was carried out on a cohort of 73 EWS patients whose clinical-pathological features are summarized in **Table 1**. Among the 35 genotyped polymorphisms, three SNPs (rs17796758, rs62468200 and rs70954368) mapping on *IGF2BP3* and one SNP (rs7930515) mapping on *SENCR* were excluded from any statistical analyses because of a low call rate.

Pairwise association analysis was performed to test the impact of the remaining variants and results are shown in **Table 2**. In details, the *IGF2BP3* rs12700421 variant was found to be significantly less frequent in EWS patients than in the control group. Specifically, heterozygote genotype led to a reduced risk of developing EWS [OR_het_ = 0.47 (95% CI 0.24-0.91)]. A similar trend was observed for the *IGF2BP3* rs13242065 variant [OR_het_ = 0.29 (95% CI 0.09-0.98)]. Instead, the adjacent rs112316332 variant showed association with increased risk of disease [OR_het_ = 1.94 (95% CI 1.08-3.49)]. The *IGF2BP3* rs146075134 variant also showed a significant association level but it was excluded from further analyses because of a deviation from Hardy-Weinberg equilibrium observed in the control-group (*P* value < 0.01). The analysis of *SENCR* polymorphisms evidenced a protective effect of the variant allele at rs11221437, rs12420823, and rs4526784 [OR_het_ = 0.48 (95% CI 0.27-0.86); OR_het_ = 0.52 (95% CI 0.3-0.92); OR_het_ = 0.5 (95% CI 0.28-0.87) respectively]. An opposite role was observed for the *SENCR* rs10893909 (*P* = 0.0092), as the variant allele of this marker increased the risk of EWS more than three times when carried in homozygosis [OR_hom_ = 3.33 (95% CI 1.35-8.19)].

**Table 2:** Case-control association analysis between EWS and polymorphisms of *IGF2BP3* and *SENCR* genes.

*IGF2BP3* SNP information					Genotype counts in controls			Genotype counts in EWS cases			Allelic association	Odds ratio (95% CI)	
chr	SNP ID	Position^a^	Alleles^b^	11		12	22	11	12	22	*P* value	OR_het_	OR_hom_
7	rs58201821	23471241	A/G	122		139	51	25	34	12	0.62	1.19 (0.68-2.11)	1.15 (0.54-2.46)
7	rs112316332	23453994	T/A	254		55	3	50	21	1	**0.03**	**1.94 (1.08-3.49)**	1.69 (0.17-16.61)
7	rs13242065	23448097	G/A	266		40	6	68	3	0	**< 0.01**	**0.29 (0.09-0.98)**	0.30 (0.02-3.38)
7	rs12533936	23447633	C/A	184		105	23	47	22	3	0.22	0.82 (0.47-1.44)	0.51 (0.15-1.77)
7	rs34033684	23445766	T/C	181		110	21	46	22	3	0.24	0.79 (0.45-1.38)	0.56 (0.16-1.97)
7	rs6953027	23442504	T/C	213		89	10	48	24	0	0.82	1.20 (0.69-2.07)	0.21 (0.01-3.64)
7	rs7782764	23436300	G/A	242		64	6	49	22	1	0.15	1.70 (0.96-3.01)	0.82 (0.10-6.99)
7	rs6971100	23433673	A/G	79		162	71	21	31	17	0.73	0.72 (0.39-1.33)	0.90 (0.44-1.84)
7	rs56041996	23432498	T/C	253		54	5	63	9	0	0.14	0.67 (0.31-1.43)	0.36 (0.02-6.65)
7	rs12700439	23432442	T/C	111		148	53	34	29	9	0.07	0.64 (0.37-1.11)	0.55 (0.25-1.24)
7	rs34543392	23419892	G/T	227		78	7	54	15	2	0.68	0.81 (0.43-1.51)	1.20 (0.24-5.94)
7	rs6651066	23416602	T/C	162		126	24	45	23	3	0.07	0.66 (0.38-1.14)	0.45 (0.13-1.56)
7	rs274034	23411179	T/A	148		134	30	31	35	5	0.89	1.25 (0.73-2.14)	0.80 (0.27-2.21)
7	rs35875486	23408895	C/T	269		42	1	65	7	0	0.34	0.69 (0.30-1.61)	1.37 (0.06-34.05)
7	rs274017	23396659	A/C	152		126	34	39	28	5	0.27	0.87 (0.51-1.49)	0.57 (0.21-1.56)
7	rs11762251	23395116	T/C	207		91	14	43	25	4	0.30	1.32 (0.76-2.30)	1.36 (0.43-4.38)
7	rs17797853	23391576	G/A	223		81	8	45	25	2	0.18	1.53 (0.88-2.65)	1.24 (0.26-6.03)
7	rs433395	23362336	G/T	103		150	59	24	32	16	0.74	0.92 (0.51-1.65)	1.16 (0.57-2.37)
7	rs3807459	23350132	T/C	96		151	65	21	35	16	0.75	1.0 (0.58-1.93)	1.13 (0.55-2.32)
7	rs12700421	23345492	C/G	209		93	10	58	12	2	**0.04**	**0.47 (0.24-0.91)**	0.72 (0.15-3.38)
7	rs10488282	23343516	T/C	207		92	13	47	24	1	0.81	1.15 (0.66-1.99)	0.34 (0.04-2.65)
7	rs76983703	23337094	G/A	150		134	28	36	27	8	0.97	0.84 (0.48-1.46)	1.19 (0.50-2.83)
7	rs17740440	23315622	C/T	246		61	5	62	10	0	0.12	0.65 (0.32-1.34)	0.36 (0.02-6.57)
7	rs199653	23312893	A/C	268		41	3	66	6	0	2.06	0.59 (0.24-1.46)	0.58 (0.03-11.30)
7	rs34414305	23311728-23311732	ATTAT/AT	139		140	33	27	29	12	0.18	1.07 (0.60-1.89)	1.87 (0.86-4.08)
7	rs3087888	23311461	C/G	270		40	2	67	5	1	0.32	0.50 (0.19-1.32)	2.02 (0.18-22.55)
*SENCR* SNP information					Genotype counts in controls			Genotype counts in EWS cases			Allelic association	Odds ratio (95% CI)	
chr	SNP ID	Position^a^	Alleles^b^	11		12	22	11	12	22	*P* value	OR_het_	OR_hom_
11	rs10893909	128695139	C/T	176		120	17	28	29	9	**< 0.01**	1.52 (0.86-2.68)	**3.33 (1.35-8.19)**
11	rs11221437	128694832	C/A	105		165	43	33	25	14	0.47	**0.48 (0.27-0.86)**	1.04 (0.51-2.13)
11	rs12420823	128693497	C/T	112		149	52	36	25	10	0.05	**0.52 (0.30-0.92)**	0.60 (0.28-1.30)
11	rs4526784	128693142	G/C	127		150	36	41	24	8	0.06	**0.50 (0.28-0.87)**	0.69 (0.30-1.60)

a UCSC Genome Browser assembly ID: hg38.

b Major allele is provided first.

chr, chromosome; SNP ID, single nucleotide polymorphism code at NCBI dbSNP; MAF, minor allele frequency; CI, confidence interval, OR_het_, odds ratio for heterozygote; OR_hom_, odds ratio for homozygote.

Bold type indicates significant association level (*P* < 0.05).

To verify if the level of association varies in relation to patient sex or age at disease onset, stratified data were considered (**Tables 3**, **4**). Females were found to be more prone than males to incur in EWS when carrying the *IGF2BP3* rs34033684 variant [OR = 3.38 (95% CI 1.44-7.94)]. An increased risk for females [OR = 2.48 (95% CI 1.28-4.82)] was also observed for the *SENCR* rs10893909 variant allele (**Table 3**). Of note, when females carry the variant at both rs34033684 and rs10893909, their risk to develop EWS is further increased [OR = 4.43 (95% CI 1.11-19.01)]. In patients with a later onset of EWS (> 14 years) a significantly lower frequency of the rare allele was observed both for *IGF2BP3* rs13242065 and rs76983703 [OR = 0.19 (95% CI 0.04-0.78) and OR = 0.29 (95% CI 0.11-0.74), respectively] (**Table 4**).

**Table 3:** Association analysis with data stratified by patient sex; only most significant data are reported.

Gene	SNP ID	Alleles^a^	Group	Variant freq.	*P* value	OR (95% CI)
*IGF2BP3*	rs34033684	T/C	Controls	0.24	*ref*	*ref*
			Males	0.13	0.02	0.47 (0.26-0.87)
			Females	0.34	0.15	1.61 (0.84-3.07)
			Males	0.13	*ref*	*ref*
			Females	0.34	**< 0.01**	**3.38 (1.44-7.94)**
*SENCR*	rs10893909	C/T	Controls	0.25	*ref*	*ref*
			Males	0.32	0.13	1.44 (0.90-2.30)
			Females	0.45	**< 0.01**	**2.48 (1.28-4.82)**
			Males	0.32	*ref*	*ref*
			Females	0.45	0.16	1.73 (0.80-3.74)

Male and female subgroups were compared with control group and between each other. ^a^Major allele first.

SNP ID, single nucleotide polymorphism code at NCBI dbSNP; freq. frequency; ref, reference; OR, odds ratio; CI, confidence interval.

Bold type indicates significant association level (*P* < 0.05).

**Table 4:** Association analysis with data stratified by age at EWS diagnosis; only most significant data are reported.

Gene	SNP ID	Alleles^a^	Group	Variant freq.	*P* value	OR (95% CI)
*IGF2BP3*	rs13242065	G/A	Controls	0.08	*ref*	*ref*
			≤ 14	0.04	0.52	0.52 (0.07-3.97)
			> 14	0.02	**0.01**	**0.19 (0.04-0.78)**
			≤ 14	0.04	*ref*	*ref*
			> 14	0.02	0.39	0.36 (0.03-4.1)
*IGF2BP3*	rs76983703	G/A	Controls	0.3	*ref*	*ref*
			≤ 14	0.54	0.02	2.74 (1.16-6.45)
			> 14	0.26	0.31	0.79 (0.51-1.24)
			≤ 14	0.54	*ref*	*ref*
			> 14	0.26	**< 0.01**	**0.29 (0.11-0.74)**

Early and late onset subgroups were compared with control group and between each other. ^a^Major allele first.

SNP ID, single nucleotide polymorphism code at NCBI dbSNP; freq. frequency; ref, reference; OR, odds ratio; CI, confidence interval.

Bold type indicates significant association level (*P* < 0.05).

The multipoint association analysis confirmed the role of the *IGF2BP3* genetic region that includes rs112316332 and rs13242065 in influencing the risk of EWS development (**Supplementary Table 1**). Significant *P* value levels were also obtained when the *IGF2BP3* rs58201821, rs12533936, rs34033684, and rs6953027 SNPs, which map close to the rs112316332 and rs13242065 SNPs, were considered in the haplotype analysis. Both over- or under-represented haplotypes were found in EWS patients. Notably, haplotypes including four, five, or six SNPs had a higher level of association compared to the effect produced by single allele in pairwise analysis.

### Impact of the *IGF2BP3* SNPs on the risk of EWS progression

**Figure 1:**
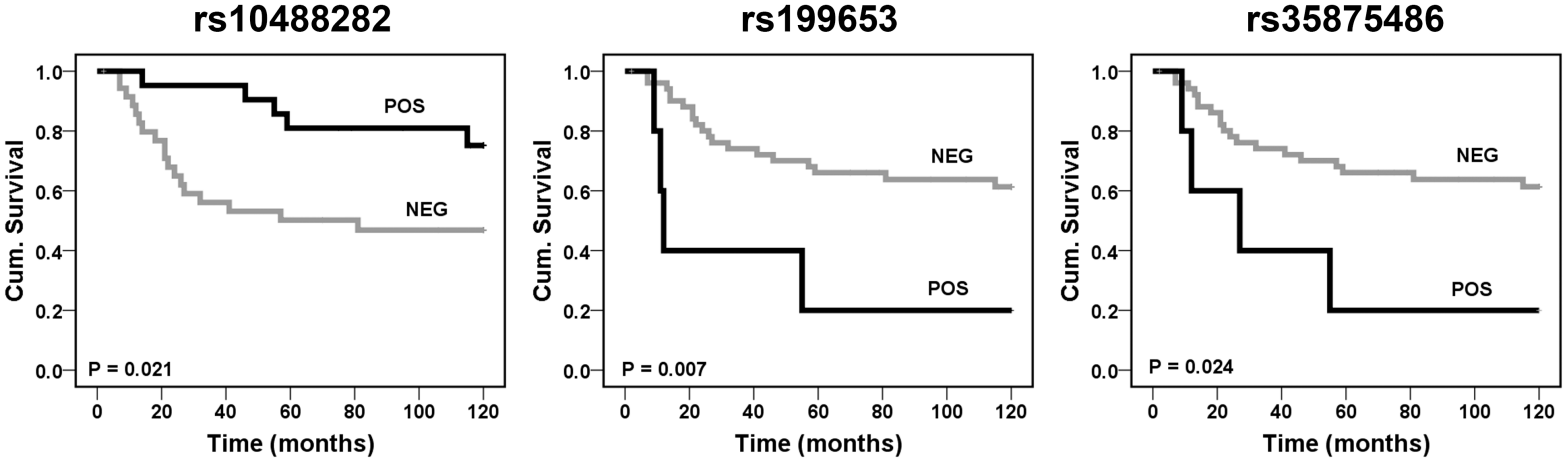
Prognostic impact of the *IGF2BP3* rs10488282, rs199653, and rs35875486 variants according to Kaplan-Meier curves and log-rank test. EWS patients were classified for the presence (POS) or absence (NEG) of the variant. Overall survival (OVS) was considered.

To search for the possibility that *IGF2BP3* or *SENCR* SNPs impact on the probability for patients to have a different outcome, we stratified patients according to the presence (censored as POS) or absence (censored as NEG) of the variant allele at the 30 previously considered SNPs (26 in *IGF2BP3* and 4 in *SENCR*, respectively). In order to limit possible drawbacks related to the presence of metastasis at diagnosis, an event known to be associated to a worse prognosis (31), we limited our analysis to 58 patients with primary, localized tumor homogeneously treated in a single Institution (**Table 1**, Dataset II). Kaplan-Meier curves and log-rank test performed on *IGF2BP3* polymorphisms showed that the absence of the variant allele at rs10488282 and the presence at rs199653 or rs35875486 were significantly associated with a worse OVS at 120 months (**Figure 1**). Multivariate analysis was performed for the three variables identified by univariate analysis and confirmed the prognostic value of the absence of the variant allele at *IGF2BP3* rs10488282 (**Table 5**) as an independent factor of worse outcome.

**Table 5:** Cox proportional-hazards regression multivariate analysis for *IGF2BP3* SNPs associated with OVS after univariate analysis in the dataset of 58 EWS patients.

Variant alleles associated with poor OVS	HR	95% CI	*P* value
rs10488282-C: NEG	2.92	1.08-7.92	**0.03**
rs199653-C: POS	4.22	0.33-52.63	0.27
rs35875486-T: POS	1.18	0.09-14.72	0.90

NEG, negative; POS, positive; HR, Hazard ratio; OVS, overall survival. Bold type indicates significant association level (*P* < 0.05).

## Discussion

Susceptibility to the development of sporadic tumors is based on a complex interplay that includes various genetic and environmental factors whose degree of influence depends on the type of cancer. In pediatric cancer etiology, the genetic contribution is predominant over the environmental one. In EWS, the peak of incidence in the second decade of life draws attention to genetic predisposition rather than to environmental repercussion for the disease onset. The identification of EWS predisposing genetic factors can lead to clinical benefits for patients, highlighting new oncogenic pathways that may be useful either for the molecular diagnosis or for better therapy. Besides wide-scale approaches, an alternative option to identify germinal predisposing factors is to deeply investigate genes already known to be involved in the biology of cancer disease.

Based on this approach, our study considers *IGF2BP3* and *SENCR* as candidate genes for searching susceptibility genetic factors to EWS. To the best of our knowledge, this is the first study linking germline genetic variants of *IGF2BP3* and of its putative modulator *SENCR* to the risk of EWS development, further supporting the concept that heritable factors can influence susceptibility to EWS (11–18).

Nominal level of significance in pairwise association analysis was obtained with three *IGF2BP3* SNPs (rs12700421, rs13242065 and rs112316332). The polymorphisms rs13242065 and rs112316332 were located in a gene region where multipoint association analysis provided evidence of association with different haplotypes. This region, bounded by rs58201821 and rs6953027, spans 29 Kbp across 5’-UTR and the second intron of the gene. According to ENCODE Registry of candidate cis-Regulatory Elements (cCREs) hosted in UCSC Genome Browser on Human GRCh38/hg38 Assembly, such region includes two cCREs showing a promoter-like signature proximal to a transcription start site (EH38E2540316 and EH38E2540292), and several predicted proximal and distal enhancers, suggesting for a potential regulatory function. The relevance of the region spanning across 5’-UTR and the second intron of *IGF2BP3* for EWS predisposition was proved also when patients were stratified for sex (rs34033684) or age (rs13242065). Our finding corroborates the hypothesis that susceptibility factors act differently in females than in males and may influence the age of EWS occurrence. In addition to *IGF2BP3* polymorphisms, our study also highlighted a potential value for *SENCR* genetic variants in EWS predisposition. All the four polymorphisms evaluated for *SENCR* were found to be significantly associated with a different risk to develop EWS and should be considered as inherited susceptibility factors of the disease. Although genetic variants in lncRNAs have been implicated as potential biomarkers in prediction of complex diseases (32), the genetic association between lncRNAs and EWS has yet to be explored. While the rs4526784 maps in the second exon of *SENCR* gene (http://genome.ucsc.edu/index.html) and may act by affecting the lncRNA sequence, the rs10893909, rs11221437 and rs12420823 map on the first intron of the gene and very likely influence EWS susceptibility in an indirect manner. For example, the rs10893909 and rs11221437 are located in regulatory regions annotated as proximal enhancer-like signature in ENCODE (EH38E1581272 and EH38E1581271, respectively) while according to the JASPAR database of transcription factor binding profiles (33) both the two variants disrupt transcription factor (TF) binding motifs. In particular, the rs10893909 variant was reported to disrupt a transcription factor binding motif with predicted affinity for several TFs, including NRF1 and KLF15 that were shown to cooperate with EWS-FLI1 (34). Likewise, the rs11221437 modifies a transcription factor binding motif with predicted affinity for CTCF, a TF involved in many cellular processes including the regulation of the transcriptional state-dependent 3D organization of the chromatin (35).

In addition, we demonstrate for the first time that three allelic variants of *IGF2BP3* may affect EWS patient’s outcome. Particularly the absence of the C allele at rs10488282 SNP was confirmed as an independent factor of prognosis at multivariate analysis, being associated with a poor survival for patients with localized EWS. Although mechanistic studies are needed to explain this observation, our findings support the hypothesis that genetic variants in the *IGF2BP3* gene may significantly affect the progression of EWS. Considering the limits related to the low number of patients here considered and the rarity of the tumor, we offer this evidence to the scientific community for more extensive validation studies. Comprehensive genomic and epigenomic profiling has revealed that epigenetic factors likely play a critical role in EWS initiation and progression (5). RNA-binding proteins, along with microRNAs and lncRNAs, which dictate the entire RNA life cycle from alternative splicing to nuclear export, transcript storage, stabilization, subcellular localization and degradation [for a review, please consider (36)], may thus represent major regulators of tumor onset and progression. Over the past few years, studies have increasingly documented the contribution of IGF2BP3 to fundamental processes in cancer biology, such as cell growth, migration, and the response to drugs. Indeed, many tumor types upregulate *IGF2BP3* compared to normal tissues but very limited information regarding the molecular regulatory mechanisms responsible for human IGF2BP3 expression is available [for a review see (21)]. Here we focused on *SENCR*, a gene coding for a lncRNA, recently found to play a critical role in the proliferation and migration of vascular smooth muscle cells (37), which may influence gene expression through multiple mechanisms, including interaction with RNA-binding proteins. The role and mechanism of action of the lncSENCR in malignant tumors remains largely unexplored. Our study supports deeper investigation on this lncRNA as a factor influencing cancer susceptibility.

## Data availability statement

The original contributions presented in the study are included in the article/**Supplementary Material**. Further inquiries can be directed to the corresponding authors.

## Ethics statement

Ethical committee approval was obtained from the Comitato Etico di Area Vasta Emilia Centro (Codice CE AVEC 505/2019/Sper/IOR). The study was conducted in accordance with the Declaration of Helsinki ethical guidelines, and patient informed consent for research use of biobanking material was obtained.

## Author contributions

MM, CZ, and KS: conception and design. MM, CM, LS, AP, PDS, CF, and MP: acquisition and analysis of data. MM, CZ, and KS: drafting the manuscript. MM and KS: study supervision. All authors contributed to the article and approved the submitted version.

## Funding

The research leading to these results has received funding from AIRC under IG 2019 — ID. 22805 project — P.I. Scotlandi Katia, and from Ricerca Fondamentale Orientata (RFO, University of Bologna) to Zucchini Cinzia and Martinelli Marcella. The materials presented and views expressed herein are the responsibility of the authors only. The sponsor takes no responsibility for any use of the information presented herein. None of the funders played a role in study design; in the collection, analysis, and interpretation of data; in the writing of the manuscript; and in the decision to submit the paper for publication.

## Acknowledgments

We thank the patients and their family for supporting this study. We thank Dr. Vilma Mantovani and Dr. Carlotta Cristalli for their technical support during experimental procedure of genotyping at CRBA (Applied Biomedical Research Center, S. Orsola-Malpighi Polyclinic, Bologna, Italy). We thank Dr. Marika Sciandra (Laboratory of Experimental Oncology, IRCCS Istituto Ortopedico Rizzoli, Bologna, Italy) for her technical support. The authors are grateful to Muscolo Skeletal Tumor Biobank-Biobanca dei Tumori Muscoloscheletrici (Biotum)—member of the CRB-IOR—which provided us the biological samples.

## Conflict of interest

The authors declare that the research was conducted in the absence of any commercial or financial relationships that could be construed as a potential conflict of interest.

## Publisher’s note

All claims expressed in this article are solely those of the authors and do not necessarily represent those of their affiliated organizations, or those of the publisher, the editors and the reviewers. Any product that may be evaluated in this article, or claim that may be made by its manufacturer, is not guaranteed or endorsed by the publisher.
