# Supplementary material for: Polymorphic variants of IGF2BP3 and SENCR have an impact on predisposition and/or progression of Ewing sarcoma
Source: Front Oncol. 2022 Oct 21;12:968884. doi: 10.3389/fonc.2022.968884 (PMC9634078; doi:10.3389/fonc.2022.968884)
Supplement: Supplementary file 1 [file Table_1.doc]

|  |  |  |  |  |  |  |  |  |  |  |
| --- | --- | --- | --- | --- | --- | --- | --- | --- | --- | --- |
| **TABLE S1 *IGF2BP3* haplotypes showing association with EWS.** | | | | | | | |  |  |  |
| **rs58201821** | **rs112316332** | **rs13242065** | **rs12533936** | **rs34033684** | **rs6953027** | **Cases** | **Controls** | **Frequency in cases** | **Frequency in controls** | ***P* value** |
|  |  |  |  |  |  |  |  |  |  |  |
| A | T | A | C | T | T | 2 | 50 | 0.02 | 0.09 | 0.005 |
| A | A | G | C | T | T | 20 | 53 | 0.16 | 0.10 | 0.035 |
|  |  |  |  |  |  |  |  |  |  |  |
| - | T | A | C | T | T | 2 | 50 | 0.02 | 0.09 | 0.005 |
|  |  |  |  |  |  |  |  |  |  |  |
| - | - | A | C | T | T | 3 | 50 | 0.02 | 0.09 | 0.012 |
| - | - | G | C | T | T | 82 | 282 | 0.62 | 0.49 | 0.006 |
|  |  |  |  |  |  |  |  |  |  |  |
| A | T | A | C | T | - | 2 | 50 | 0.02 | 0.09 | 0.005 |
| G | T | G | C | T | - | 28 | 83 | 0.22 | 0.14 | 0.040 |
|  |  |  |  |  |  |  |  |  |  |  |
| - | T | A | C | T | - | 2 | 50 | 0.02 | 0.09 | 0.005 |
|  |  |  |  |  |  |  |  |  |  |  |
| - | - | A | C | T | - | 3 | 50 | 0.02 | 0.09 | 0.012 |
| - | - | G | C | T | - | 105 | 390 | 0.79 | 0.67 | 0.006 |
|  |  |  |  |  |  |  |  |  |  |  |
| A | T | A | C | - | - | 2 | 52 | 0.02 | 0.08 | 0.004 |
| A | A | G | C | - | - | 21 | 57 | 0.15 | 0.09 | 0.032 |
|  |  |  |  |  |  |  |  |  |  |  |
| - | T | A | C | - | - | 2 | 52 | 0.01 | 0.08 | 0.004 |
|  |  |  |  |  |  |  |  |  |  |  |
| - | - | A | C | - | - | 3 | 52 | 0.02 | 0.08 | 0.010 |
| - | - | G | C | - | - | 109 | 421 | 0.78 | 0.67 | 0.015 |
|  |  |  |  |  |  |  |  |  |  |  |
| A | T | A | - | - | - | 2 | 52 | 0.02 | 0.08 | 0.004 |
| A | A | G | - | - | - | 21 | 57 | 0.15 | 0.09 | 0.031 |
|  |  |  |  |  |  |  |  |  |  |  |
| - | T | A | - | - | - | 3 | 52 | 0.02 | 0.08 | 0.010 |
| - | A | G | - | - | - | 22 | 61 | 0.16 | 0.10 | 0.041 |
|  |  |  |  |  |  |  |  |  |  |  |
| A | T | - | - | - | - | 61 | 325 | 0.43 | 0.52 | 0.040 |
| A | A | - | - | - | - | 23 | 57 | 0.16 | 0.09 | 0.015 |
|  |  |  |  |  |  |  |  |  |  |  |
|  |  |  |  |  |  |  |  |  |  |  |
